# Tumor boards and multidisciplinary cancer treatment: history, evidence, impact, and future perspectives

**DOI:** 10.1590/1516-3180.2026.1446.24082026

**Published:** 2026-10-23

**Affiliations:** I Thoracic Surgeon, graduate of the Instituto do Coração (InCor), Hospital das Clínicas (HCFMUSP), Faculdade de Medicina, Universidade de São Paulo (USP), São Paulo (SP), Brazil. Fellow in Thoracic Oncology, Instituto do Câncer do Estado de São Paulo (ICESP), Instituto do Coração (InCor), Hospital das Clínicas (HCFMUSP), Faculdade de Medicina, Universidade de São Paulo (USP), São Paulo (SP), Brazil.; II Thoracic Surgeon, Instituto do Câncer do Estado de São Paulo, Hospital das Clínicas, Faculdade de Medicina, Universidade de São Paulo (USP), São Paulo (SP), Brazil. Thoracic Surgeon, Hospital Sírio-Libanês, and member of the Núcleo Avançado de Tórax do Hospital Sírio-Libanês, São Paulo (SP), Brazil.; III Full Professor, Thoracic Surgery Program, Instituto do Coração, Hospital das Clínicas, Faculdade de Medicina, Universidade de São Paulo (USP), São Paulo (SP), Brazil; Director, Scientific Department, Associação Paulista de Medicina (APM), São Paulo (SP), Brazil.

Currently, cancer treatment is one of the most complex types of treatment in medicine. The range of treatment modalities—surgery, radiotherapy, systemic therapies, and precision medicine—is constantly evolving, and no single specialist can fully master them all. In this sense, *tumor boards* have become established as regular forums in which professionals from different specialties discuss cases and make shared decisions regarding clinical management. More than clinical meetings, these boards represent a culture of multidisciplinary decision-making that places the patient at the center and reduces the risk of decisions being made that are guided by isolated perspectives.^1,2^

## Composition of tumor boards

The effectiveness of a tumor board begins with defining the team. The most common models feature a crucial core comprising a surgeon, medical oncologist, radiation oncologist, interventional radiologist, and pathologist—that is, specialists directly involved in defining the treatment plan.^1^ Support professionals such as nurse navigators, nutritionists, psychologists, pharmacists, and geneticists provide a more holistic view of the patient beyond the cancer treatment itself.^2^ These meetings are organized according to the specific tumor sites, such as the thorax, head and neck, esophagus, and breast, and they encompass all subspecialties of oncology.

Importantly, tumor boards cover the treatment of the disease itself as well as the entire journey of the patient. This includes diagnosis, staging, treatment, and follow-up, and also the management of potential treatment-related complications.

## Rationale for implementation

The conceptual framework was established in a systematic review conducted by Wright et al.,^1^ in which practice standards were defined for tumor boards, including minimum team composition, meeting frequency, case selection, documentation, and assessment. In a seminal review in *The Lancet Oncology*, Fleissig et al.^2^ critically evaluated the effectiveness of multidisciplinary teams, highlighting both the benefits and heterogeneity of the available evidence. More recently, an umbrella review in which five systematic reviews were analyzed confirmed the impact of tumor boards across three dimensions: diagnosis, with greater staging accuracy and diagnostic precision in 89% to 93.5% of cases; treatment, with changes in management and greater adherence to clinical guidelines; and survival, with documented improvements for colorectal, head and neck, breast, esophageal, and lung cancers, although outcomes were heterogeneous across the studies.^3^

In the comparative interventional cohort study conducted by Kesson et al.,^4^ involved 13,722 women with breast cancer in Scotland, introducing multidisciplinary care reduced breast cancer–specific mortality by 18% (HR = 0.82; 95%CI = 0.74–0.91), with less variation in outcomes among hospitals. In a recent meta-analysis, a 14% reduction in the relative risk of mortality was quantified among patients whose cases were assessed by tumor boards.^5^ In Brazil, a prospective study conducted at Hospital Alemão Oswaldo Cruz documented a change in therapeutic management in 25.7% of 413 cases discussed.^6^ Furthermore, a Grupo Brasileiro de Oncologia Torácica/Latin America Cooperative Oncology Group (GBOT/LACOG) survey of 206 professionals revealed that 91.6% believed that the use of tumor boards improved outcomes. However, 21.1% of physicians and 32.7% of non-physicians reported feeling hesitant to voice their opinions, serving as a reminder that the forum's effectiveness depends both on its structure and on an institutional culture that provides a safe environment for diverse opinions to be heard and discussed.^7^

## Lung cancer and thoracic surgery

In lung cancer, the choice among surgical resection, stereotactic radiotherapy, systemic therapies, and multimodal approaches depends on accurate staging and the discussion of borderline cases—decisions best made as a team. The introduction of a multidisciplinary team for patients with inoperable non-small cell lung cancer was evaluated in a single-center, pre–post intervention study: Forrest et al.^8^ observed an increase in chemotherapy use, from 7% to 23% (P < 0.001), and a doubling of median survival, from 3.2 to 6.6 months (P < 0.001). In a prospective cohort study reported in *The Annals of Thoracic Surgery*, Schmidt et al.^9^ showed that the *weekly thoracic tumor board* changed the treatment plan in 40% of lung cancer cases and 26% of esophageal cancer cases, with a 97% adherence to the recommendations. A 2024 systematic review and meta-analysis of 20 studies confirmed that presenting cases at multidisciplinary team meetings increased histological confirmation rates (odds ratio = 3.01; 95%CI = 2.30–3.95), the likelihood of surgical treatment (odds ratio = 2.01; 95%CI = 1.29–3.12), and overall survival (hazard ratio = 0.63; 95%CI = 0.55–0.72).^10^

## The Instituto do Câncer do Estado de São Paulo experience

The Instituto do Câncer do Estado de São Paulo is the largest public oncology center in Latin America. It began operating in 2008 using a nonprofit management model known as an Organização Social de Saúde (Social Health Organization), and under the technical and scientific coordination of the Faculdade de Medicina da Universidade de São Paulo, it became a state-level oncology referral center within the Brazilian Unified Health System.^11^ The institute is structured into integrated clinics according to tumor site, with teams that bring together a medical oncologist, surgeon, and other medical specialists. Weekly institutional tumor boards operate within this framework, and during their meetings—in addition to the specialists mentioned above—radiologists, interventional radiologists, and pathologists discuss the most complex cases. This experience was documented in a retrospective cohort study of 321 patients with advanced laryngeal squamous cell carcinoma with stage T3/T4a disease treated between 2009 and 2019. Before the formal implementation of the tumor board in 2014, cumulative survival was clearly higher in the surgical group (61.1% vs. 28.4%; P = 0.002). After the weekly meeting was implemented, the outcomes of total laryngectomy and chemoradiotherapy became comparable, and chemoradiotherapy transitioned from being an independent risk factor for death to a protective factor. This provides evidence, generated within a public health system, that joint decision-making improves overall patient outcomes.^12^

## Future directions

Although the evidence has established the value of tumor boards, it has also revealed gaps. Current challenges include hierarchical barriers that inhibit participation (21.1% of physicians and 32.7% of non-physicians reported feeling hesitant to voice their opinions), insufficient documentation and auditing, variable adherence to tumor board recommendations, and a lack of systematic outcome indicators. Thus, future progress relies on three key areas: standardization and auditing of structures, processes, and outcomes;^1^ expansion of the use of hybrid and virtual formats, accelerated by the pandemic and preferred by 52.7% of Brazilian professionals^7^; and incorporation of artificial intelligence and precision medicine, as well as improved documentation and decision support systems. These should always serve as tools to support clinical judgment. The tumor board model is therefore a scientifically effective system designed to improve overall outcomes from the beginning of the patient journey, and it is already well established in Brazil, as demonstrated by the documented Instituto do Câncer do Estado de São Paulo experience.

## References

[1] Wright FC, De Vito C, Langer B, Hunter A (2007). Expert Panel on Multidisciplinary Cancer Conference Standards. Multidisciplinary cancer conferences: a systematic review and development of practice standards. Eur J Cancer.

[2] Fleissig A, Jenkins V, Catt S, Fallowfield L (2006). Multidisciplinary teams in cancer care: are they effective in the UK?. Lancet Oncol.

[3] Specchia ML, Frisicale EM, Carini E (2020). The impact of tumor board on cancer care: evidence from an umbrella review. BMC Health Serv Res.

[4] Kesson EM, Allardice GM, George WD, Burns HJ, Morrison DS (2012). Effects of multidisciplinary team working on breast cancer survival: retrospective, comparative, interventional cohort study of 13,722 women. BMJ.

[5] Di Pilla A, Cozzolino MR, Mannocci A (2022). The impact of tumor boards on breast cancer care: evidence from a systematic literature review and meta-analysis. Int J Environ Res Public Health.

[6] Costa MA, Teixeira CHA, Peixoto RDA (2020). Multidisciplinary tumor boards: a prospective study of the impact on patient management in a community-based Brazilian cancer center. J Clin Oncol.

[7] Menezes TU, Serra MMP, Barrios CH (2023). Results of a survey study on health professionals’ perceptions of tumor boards in Brazil. Future Oncol.

[8] Forrest LM, McMillan DC, McArdle CS, Dunlop DJ (2005). An evaluation of the impact of a multidisciplinary team, in a single centre, on treatment and survival in patients with inoperable non-small-cell lung cancer. Br J Cancer.

[9] Schmidt HM, Roberts JM, Bodnar AM (2015). Thoracic multidisciplinary tumor board routinely impacts therapeutic plans in patients with lung and esophageal cancer: a prospective cohort study. Ann Thorac Surg.

[10] Stirling RG, Harrison A, Huang J (2024). Multidisciplinary meeting review in nonsmall cell lung cancer: a systematic review and meta-analysis. Eur Respir Rev.

[11] Madi MR, Cerri GG (2018). Organization of the cancer network in SUS: evolution of the care model. Clinics.

[12] Rodrigues MG, Ramos DM, de Matos LL (2025). Different treatment modalities for advanced laryngeal cancer: improvement of oncological outcomes after tumor board implementation. Clinics.

